# MAPK-activated protein kinase 2-deficiency causes hyperacute tumor necrosis factor-induced inflammatory shock

**DOI:** 10.1186/s12899-014-0005-1

**Published:** 2014-09-04

**Authors:** Benjamin Vandendriessche, An Goethals, Alba Simats, Evelien Van Hamme, Peter Brouckaert, Anje Cauwels

**Affiliations:** Inflammation Research Center, VIB, 9052 Ghent, Belgium; Department of Biomedical Molecular Biology, Ghent University, 9000 Ghent, Belgium; Bio Imaging Core, Inflammation Research Center, VIB, 9052 Ghent, Belgium; Cytokine Receptor Lab - Department of Medical Protein Research, VIB, 9000 Ghent, Belgium

**Keywords:** MK2, Inflammatory shock, Tumor necrosis factor, Reactive oxygen species, Actin cytoskeleton, Endothelial permeability, Cecal ligation and puncture

## Abstract

**Background:**

MAPK-activated protein kinase 2 (MK2) plays a pivotal role in the cell response to (inflammatory) stress. Among others, MK2 is known to be involved in the regulation of cytokine mRNA metabolism and regulation of actin cytoskeleton dynamics. Previously, MK2-deficient mice were shown to be highly resistant to LPS/d-Galactosamine-induced hepatitis. Additionally, research in various disease models has indicated the kinase as an interesting inhibitory drug target for various acute or chronic inflammatory diseases.

**Results:**

We show that in striking contrast to the known resistance of MK2-deficient mice to a challenge with LPS/D-Gal, a low dose of tumor necrosis factor (TNF) causes hyperacute mortality via an oxidative stress driven mechanism. We identified *in vivo* defects in the stress fiber response in endothelial cells, which could have resulted in reduced resistance of the endothelial barrier to deal with exposure to oxidative stress. In addition, MK2-deficient mice were found to be more sensitive to cecal ligation and puncture-induced sepsis.

**Conclusions:**

The capacity of the endothelial barrier to deal with inflammatory and oxidative stress is imperative to allow a regulated immune response and maintain endothelial barrier integrity. Our results indicate that, considering the central role of TNF in pro-inflammatory signaling, therapeutic strategies examining pharmacological inhibition of MK2 should take potentially dangerous side effects at the level of endothelial barrier integrity into account.

## Background

MAP kinase (MAPK)-activated protein kinase 2 (MK2) is involved in very diverse cellular processes ranging from cell migration, to reorganization of the cytoskeleton, inflammation and apoptosis. MK2 is located downstream from p38 MAPK, a major transducer of cell stress responses, such as heat shock, bacterial lipopolysaccharide (LPS), tumor necrosis factor (TNF) and IL-1β (reviewed in [1,2]). In response to an inflammatory stressor such as LPS, p38 will phosphorylate and activate a number of effectors, including MK2. The resulting p38-MK2 complex will translocate from the nucleus to the cytoplasm, followed by MK2-mediated phosphorylation of target proteins (reviewed in [3]). Because of this requirement for co-translocation of p38 and MK2, MK2-deficient mice are also partially p38 MAPK-deficient [4].

One of the better characterized MK2-mediated processes is the post-transcriptional regulation of pro-inflammatory cytokine production [5], including but not limited to TNF [6], IL-6 [7,8], IFN-γ [9], and possibly IL-1β [10]. Tristetraprolin (TTP), an mRNA-binding zinc-finger protein, destabilizes many mRNAs by binding to AU-rich elements (AREs) in their 3’ untranslated region [11], and subsequently targeting them to exosomes or proteasomes [12,13]. MK2 can phosphorylate TTP, thereby reducing its affinity for the ARE [14,15] and allowing other mRNA-binding protein complexes to form that alter the dynamics of pro-inflammatory mRNA metabolism. For instance, in the case of TNF mRNA translation, human antigen R (HuR) can replace phosphorylated TTP, thereby stabilizing the mRNA and initiating translation of TNF [16]. In addition, the expression and post-translational regulation of TTP itself is also regulated by p38-MK2-dependent signal transduction, which allows intrinsic feedback control of the inflammatory response [15,16]. As a result of the dependency of many pro-inflammatory mRNAs on MK2-mediated stabilization, the expression of these cytokines in response to many inflammatory challenges is severely altered in MK2-deficient mice. This was first shown for TNF biosynthesis in response to a combined LPS/d-Galactosamine (d-Gal) challenge, a model of acute hepatitis [6]. Another target of p38 MAPK is MK3. The documented functions and expression patterns of MK2 and MK3 overlap, but MK2 is expressed at significantly higher levels, and assumed to be the dominant isoform. Consistently, TNF production in MK3-deficient mice is comparable to wild type, while expression in MK2/MK3 double-knockout mice was reduced further compared to MK2-deficient mice [17].

A second major p38/MK2-mediated function is the regulation of actin cytoskeleton dynamics through phosphorylation of the small heat shock protein (sHSP) 25 (mouse equivalent to human sHSP27) [1,18]. The actin cytoskeleton plays essential roles in many cellular processes, including regulation of cell shape, endocytosis, anchorage to cells and substrates, and signal transduction. Because of the ability of sHSPs to exist in various states of oligomerization, depending on their phosphorylation status, they can be involved in highly diverse cellular functions, including molecular chaperoning [19], modulation of the glutathione redox status [20], and regulation of actin polymerization dynamics [21].

The endothelial cell layer plays a pivotal role in the inflammatory response by regulating the passage of leukocytes and various signaling molecules, and is an important signal transduction interface. Put differently, its main function is to maintain a highly specialized and selective barrier between the blood and other tissues. During systemic inflammatory conditions, endothelial cells can become exposed to extremely high levels of inflammatory mediators, such as reactive oxygen species (ROS). When ROS-induced damage reaches critical levels or cannot be dealt with appropriately, the barrier function can become severely impaired, resulting in uncontrolled fluid leak, a dysregulated immune response, cell death, and tissue damage. Therefore, endothelial cells have evolved mechanisms to quickly reorganize their actin cytoskeleton into stress fibers that serve both as a contraction scaffold to allow a controlled increase in endothelial permeability and extravasation of leukocytes [22,23], as well as firmly anchor the cells to their neighbors and the matrix to preserve overall barrier integrity [24–26].

In the current study, we examined MK2-deficiency in a model of TNF or LPS-induced systemic inflammation, as well as cecal ligation and puncture (CLP)-induced sepsis. We identified profound and hyperacute TNF-induced mortality, in sharp contrast to the previously described resistance of MK2-deficient mice to LPS/d-Gal-induced hepatitis [6] and similar results we obtained here in an endotoxic shock model. This hyperacute phenotype appeared to be caused by the inability of MK2-deficient endothelial cells to mount a proper stress fiber response, resulting in excessive fluid leak, tissue damage and mortality. Additionally, MK2-deficient mice were also sensitized to CLP-induced mortality.

## Results

### The effect of MK2-deficiency on mortality induced by systemic inflammation or sepsis

**Figure 1:**
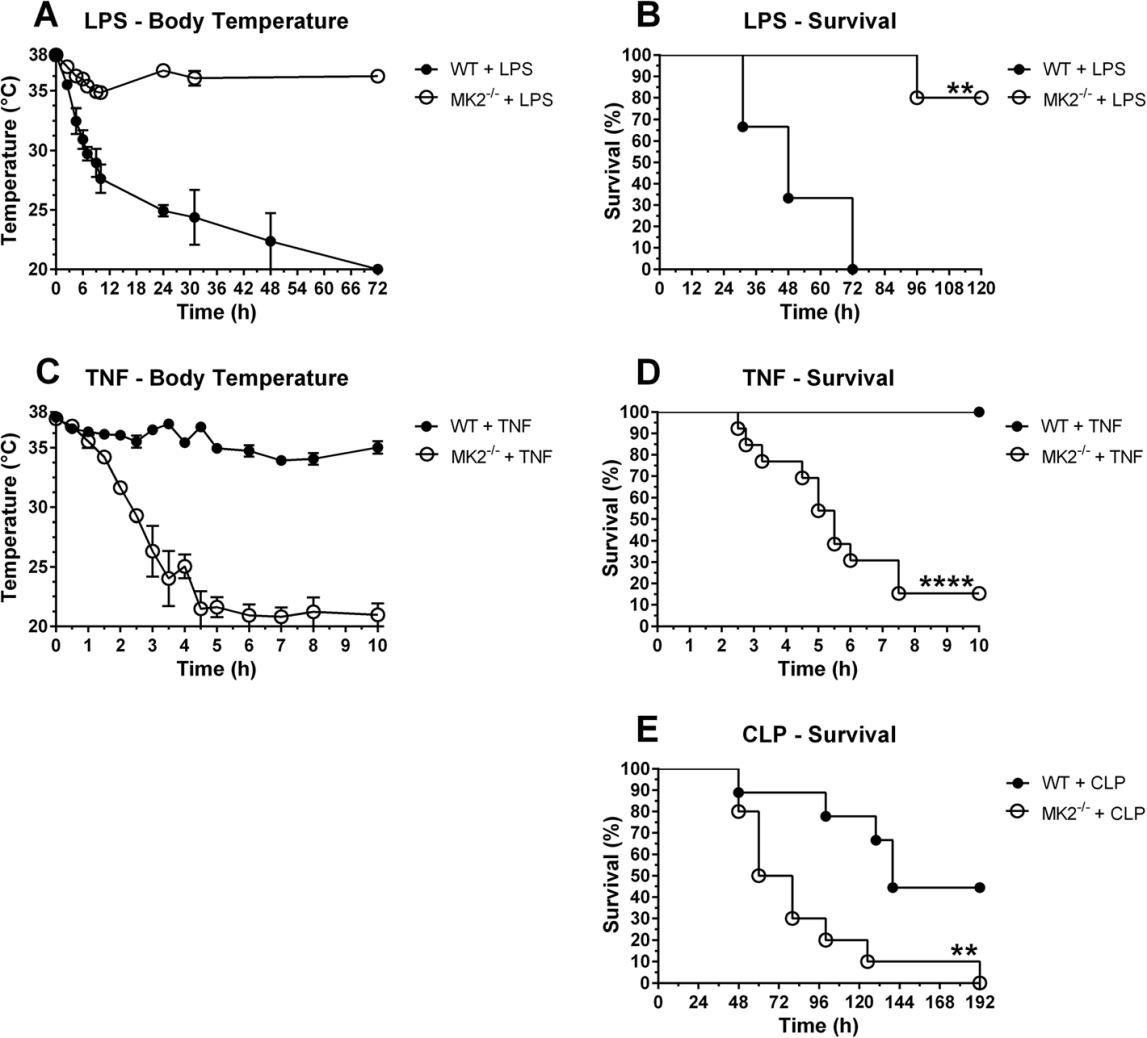
**Effect of MK2-deficiency on morbidity and mortality in systemic inflammation and sepsis. (A-B)** Body temperature and mortality for WT (n = 3) and MK2^−/−^ (n = 5) mice after i.v. injection of 10 mg/kg LPS. **(C)** Body temperature for WT (n = 5) and MK2^−/−^ (n = 9) mice after i.v. injection of 50 or 125 μg/kg TNF. Dose groups were pooled together because statistical comparison within one genotype revealed no differences. **(D)** Mortality for WT (n = 10) and MK2^−/−^ (n = 13) mice after i.v. injection of 10, 50, 125 or 250 μg/kg TNF. Dose groups were pooled together because statistical comparison within one genotype revealed no differences. **(E)** Mortality for WT (n = 9) and MK2^−/−^ (n = 10) mice after CLP surgery. Survival curves of different genotypes were compared via log-rank test. ****, p ≤ 0.0001; **, p ≤ 0.01.

MK2-deficient mice were originally shown to be resistant to LPS/d-Gal [6]. However, LPS/d-Gal causes apoptosis-dependent hepatitis and not endotoxic shock [27], as often mentioned. In order to determine the response of MK2-deficient animals to endotoxic shock, we administered a high i.v. dose of LPS and found that MK2-deficiency also confers extreme protection to LPS-induced hypothermia and mortality (Figure 1A-B). In sharp contrast to these results, MK2-deficient mice were hypersensitive to TNF-induced inflammatory shock (Figure 1C-D). Low to very low doses of TNF (10 – 250 μg/kg), which in wild type (WT) mice only induced a small and temporary drop in body temperature, caused a very fast drop in body temperature in MK2-deficient animals, followed by acute mortality 2–8 h after challenge. In addition, we examined the response of MK2-deficient animals to cecal ligation and puncture (CLP)-induced sepsis. Similarly to TNF, MK2-deficient mice were also sensitized to CLP-induced sepsis (Figure 1E).

### Acute TNF-induced inflammatory mediators and cell damage parameters

**Figure 2:**
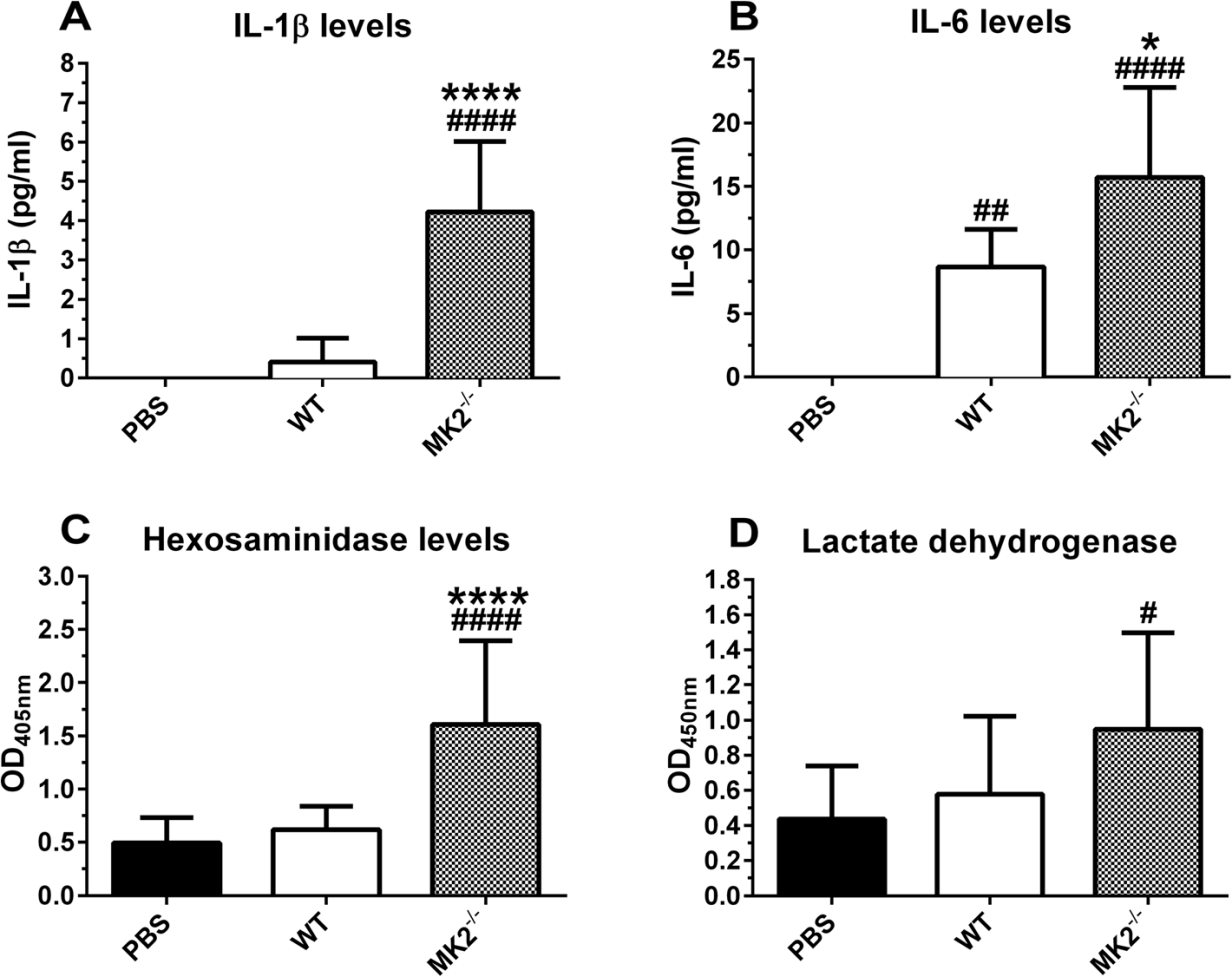
**IL-1β, IL-6, hexosaminidase and lactate dehydrogenase levels. (A-B)** Plasma IL-1β and IL-6 levels for WT and MK2^−/−^ mice, 2.5 h after i.v. injection of 450 μg/kg TNF (n^PBS^ (WT and MK2^−/−^) = 10, n^TNF^ (WT) = 5, n^TNF^ (MK2^−/−^) = 5). **(C-D)** Plasma hexosaminidase and lactate dehydrogenase plasma levels for WT and MK2^−/−^ mice, 2.5 h after i.v. injection of 450 μg/kg TNF. Pooled data from 2 separate experiments is shown (n^PBS^ (WT and MK2^−/−^) = 17, n^TNF^ (WT) = 10, n^TNF^ (MK2^−/−^) = 8). Comparisons were made between baseline (PBS) and TNF challenged animals (#), and between genotypes (*) via one-way ANOVA with Sidak’s multiple comparisons test. Error bars indicate SD. ****, p ≤ 0.0001; **, p ≤ 0.01; *, p ≤ 0.05.

IL-1β and IL-6 levels were significantly increased in MK2-deficient mice 2.5 h after TNF challenge compared to their WT counterparts (Figure 2A-B). Of note, a high dose of TNF (450 μg/kg) was used to also evoke a clear inflammatory response in WT mice. Intracellular proteins such as hexosaminidase, a lysosomal enzyme, and lactate dehydrogenase (LDH) are indicative for cellular damage and (necrotic) disintegration when detected systemically. Plasma hexosaminidase was significantly increased above baseline in MK2-deficient mice only, 2.5 h after challenge (Figure 2C). Similarly, LDH was only significantly increased for MK2-deficient animals (Figure 2D).

### The hypersensitivity of MK2-deficient mice for TNF can be prevented by antioxidant treatment

**Figure 3:**
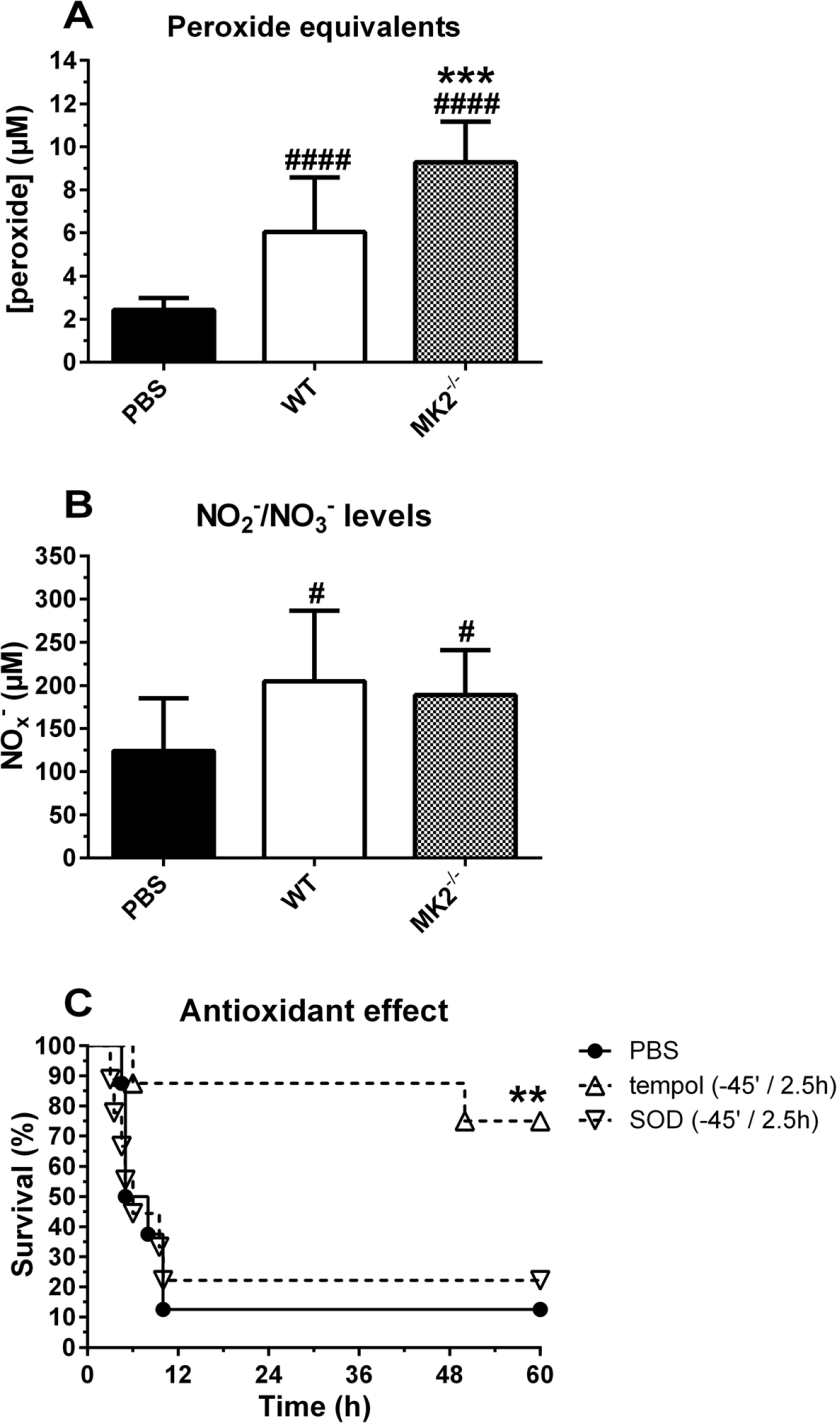
**Peroxide equivalents, NO**_**x**_^**−**^ **levels, and effect of antioxidant treatment on mortality. (A)** Plasma peroxide equivalents for WT and MK2^−/−^ mice, 2.5 h after i.v. injection of 125 μg/kg TNF. Pooled data from 2 separate experiments is shown (n^PBS^ (WT and MK2^−/−^) = 13, n^TNF^ (WT) = 11, n^TNF^ (MK2^−/−^) = 12). **(B)** Plasma NO_x_^−^ levels for WT and MK2^−/−^ mice, 2.5 h after i.v. injection of 125 μg/kg TNF. Pooled data from 2 separate experiments is shown (n^PBS^ (WT and MK2^−/−^) = 12, n^TNF^ (WT) = 7, n^TNF^ (MK2^−/−^) = 9). Comparisons were made between baseline (PBS) and TNF challenged animals (#), and between genotypes (*) via one-way ANOVA with Sidak’s multiple comparisons test. **(C)** Mortality for TNF challenged MK2^−/−^ mice after pre- and post-treatment with tempol or SOD. Survival curves of different treatment groups were compared to controls via log-rank test (*). Error bars indicate SD. ****, p ≤ 0.0001; ***, p ≤ 0.001; **, p ≤ 0.01; *, p ≤ 0.05.

Circulating peroxide equivalents, a measure for total oxidative stress exposure, were significantly higher in plasma of MK2-deficient animals compared to WT mice, already 2.5 h after injection of TNF (Figure 3A), while NO_x_^−^ levels, a measure for total nitric oxide (NO) production, were similarly increased from baseline for both genotypes (Figure 3B). We treated MK2-deficient animals with antioxidants to verify the influence of oxidative stress in the pathophysiology of the hyperacute response. The membrane-permeable superoxide dismutase (SOD) mimetic and radical scavenger tempol could significantly protect against TNF-induced mortality as a combined pre- and post-treatment, while the non-membrane permeable SOD had no effect (Figure 3C).

### TNF challenged MK2-deficient animals have a defective stress fiber response

**Figure 4:**
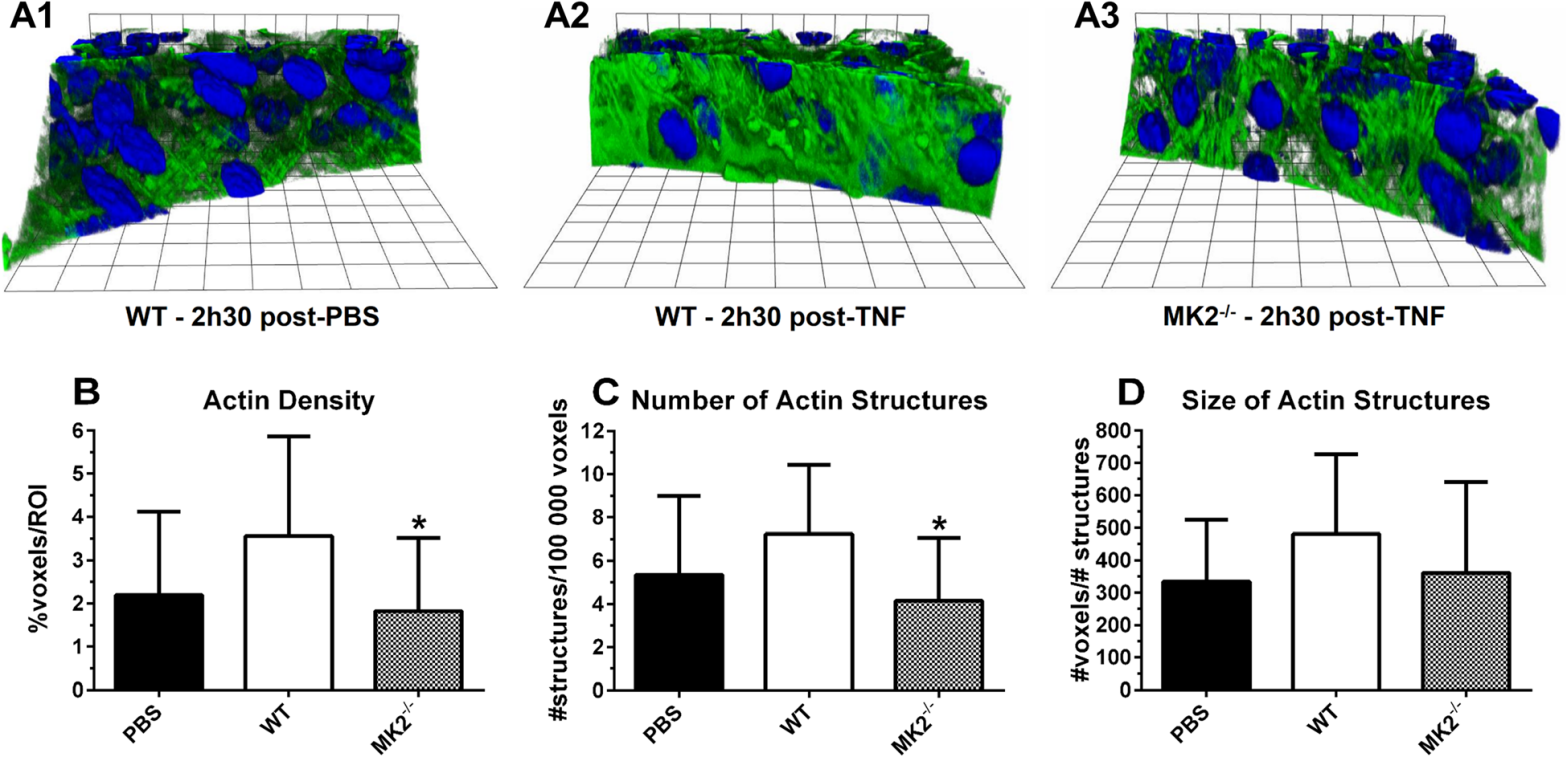
**Assessment of cytoskeletal integrity via quantification of F-actin structures and density in liver endothelial cells. (A)** Representative liver z-stacks stained for F-actin (green) and DAPI (blue), with a focus on the endothelial cell lining of a small vessel for WT and MK2^−/−^ mice, 2.5 h after i.v. injection of PBS or 450 μg/kg TNF; (n^PBS^ (WT and MK2^−/−^) = 10 × 3 vessels/animal, n^TNF^ (WT) = 5 × 3, n^TNF^ (MK2^−/−^) = 5 × 3). For every vessel, part of the endothelial cell sheet was defined as region of interest (ROI). **(B)** Actin density, expressed as the percentage of F-actin positive voxels per ROI. **(C)** The number of segmented F-actin positive structures per 100 000 voxels. **(D)** The average size of the actin structures, expressed as the total number of F-actin positive voxels per actin structure, normalized over the number of discrete actin structures. Comparisons were made between baseline (PBS) and TNF challenged animals (#), and between genotypes (*) via one-way ANOVA with Sidak’s multiple comparisons test. Error bars indicate SD. *, p ≤ 0.05.

Liver sections were stained for F-actin and 3 small vessels per section were imaged. Representative examples are shown in Figure 4A1-3. Part of the endothelial cell sheet was manually defined as a region of interest (ROI), followed by segmentation of the ROI in discrete actin structures. Next, a number of parameters were calculated: (1) the percentage of F-actin positive voxels in the ROI, as a measure for the density of the actin network (Figure 4B); (2) the number of discrete actin structures per 100 000 voxels (Figure 4C); and (3) the total number of F-actin positive voxels per actin structure normalized over the number of discrete actin structures, as an estimator of the size of the actin structures (Figure 4D). F-actin density and the number of F-actin structures were not increased from baseline in MK2-deficient mice, in contrast to WT controls, indicating a failure of the endothelial cells to mount a proper stress fiber response (Figure 4B-C). In addition, the size of the F-actin structures was lower in the TNF challenged MK2-deficient animals compared to WT but this difference was not significant (Figure 4D).

### TNF-induced vascular leak is much more pronounced in MK2-deficient mice

**Figure 5:**
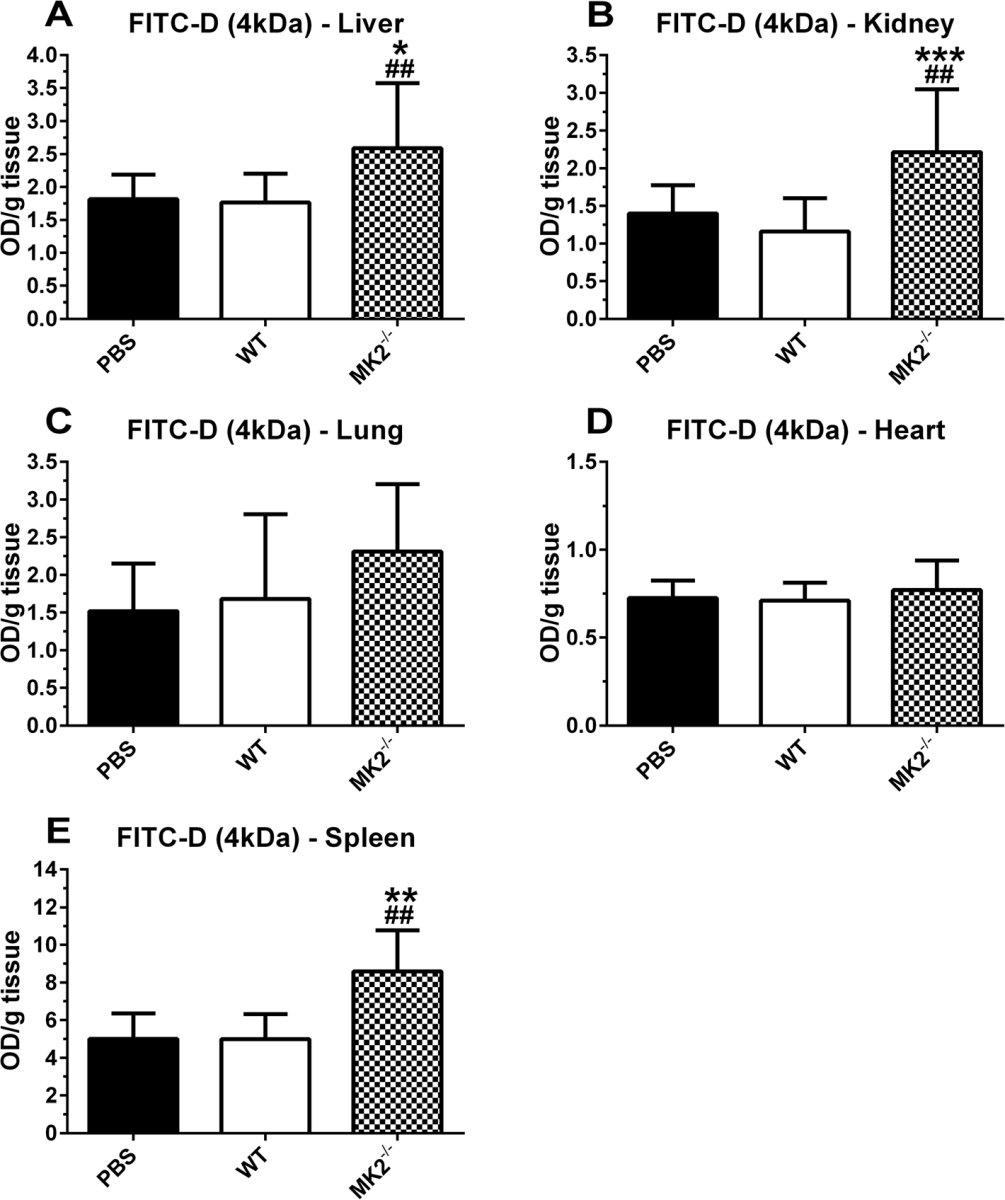
**Vascular permeability assessed by extravasation of FITC-Dextran (4 kDa).** FITC-Dextran quantification for WT and MK2^−/−^ mice, 2.5 h after i.v. injection of 450 μg/kg TNF for liver **(A)**, kidney **(B)**, lung **(C)**, heart **(D)**, and spleen **(E)**; (n = 5 × 2 for liver, kidney and lungs; n = 5 for heart and spleen). Comparisons were made between baseline (PBS) and TNF challenged animals (#), and between genotypes (*) via one-way ANOVA with Sidak’s multiple comparisons test. Error bars indicate SD. ***, p ≤ 0.001; **, p ≤ 0.01; *, p ≤ 0.05.

Vascular permeability was quantified by examining extravasation of FITC-dextran (4 kDa) and Evans Blue. The latter binds albumin with high affinity, resulting in an approximate molecular weight of 70 kDa. No significant differences in Evans blue extravasation were observed (data not shown). However, the smaller molecular weight FITC-dextran was significantly increased from baseline in liver, kidney and spleen of MK2-deficient mice, in contrast to WT controls (Figure 5A-B and E). Also in the lungs, a small increase in permeability was observed, albeit non-significant, while no effect was observed in the heart (Figure 5C-D).

## Discussion

Our results show that TNF induces a hyperacute inflammatory shock phenotype in MK2-deficient mice. A lethal TNF challenge in WT mice will cause mortality between roughly 16–24 h post-challenge (data not shown), while a low dose of TNF normally causes only mild and transient morbidity in healthy WT mice. However, in MK2-deficient mice, very low doses of TNF caused hyperacute mortality already 2–8 h post-challenge. In addition, we corroborated earlier results that demonstrated the resistance of MK2-deficient mice to LPS/d-Gal induced hepatitis [6,27], by showing that MK2-deficient mice are also resistant to endotoxic shock. The resistance of MK2-deficient mice to LPS/d-Gal has been clearly linked to reduced stability of TNF and other mRNAs encoding pro-inflammatory cytokines, via p38 MAPK/MK2/TTP-mediated signaling [7,8,14]. However, in our TNF model, IL-6 and IL-1β levels in circulation were increased more in MK2-deficient animals after the challenge, compared to WT controls. This could indicate that the hyperacute phenotype is caused by such a severe inflammatory response that, despite decreased mRNA stability of pro-inflammatory cytokines, high levels of these cytokines could still be detected in circulation. To corroborate this further, parameters indicative for (necrotic) cellular disintegration were highly increased early after TNF challenge in MK2-deficient mice, while they were not increased from baseline for WT controls that received a high dose of TNF.

We previously observed a similar hyperacute phenotype in WT mice after a combined challenge with TNF and the pan-caspase inhibitor zVAD-fmk [28]. In that case and others, ROS were found to play an important role in the pathophysiology [28,29]. Also in the current study, highly increased levels of radicals were detected after challenge with TNF in the MK2-deficient animals. Therefore, we treated these animals with the antioxidants tempol or SOD. Tempol is a membrane permeable antioxidant that dismutates superoxide catalytically and limits Fenton-mediated hydroxyl radical formation [30]. Contrary, the action of SOD is limited to the dismutation of superoxide and it is not membrane permeable. Thus, the observed difference in response could indicate that the ability of tempol to diffuse into cells and/or its broader spectrum antioxidant activity are key factors for preventing the acute TNF-induced toxicity. In any case, it indicates that ROS are an important driver of the TNF-induced pathophysiology in the absence of MK2. The small heat shock protein 25 (sHSP25) can form large oligomers that are known to be involved in the maintenance of intracellular glutathione (GSH) levels and help keep GSH in its reduced form under conditions of oxidative stress [20,31], in addition to their well-known protein chaperoning functions [32]. It is therefore tempting to speculate that the absence of MK2, which can modulate the function of sHSP25 via phosphorylation, could be the cause of the high ROS levels, resulting in tissue damage and the observed acute mortality. However, upon MK2-mediated phosphorylation the large sHSP25 oligomers dissociate into smaller oligomers and monomers [33,34], and the antioxidant capacity of sHSP25 has been clearly linked to its unphosphorylated oligomerized form [31]. Decreased sHSP25-mediated antioxidant capacity is therefore unlikely to be a dominant mechanism in the current model.

Next, we examined actin cytoskeleton dynamics, another target of MK2 via phosphorylation of sHSP25. As mentioned earlier, large sHSP25 oligomers will dissociate into smaller oligomers and monomers upon phosphorylation [33,34]. Considering that signal transduction occurs through the stress-activated p38 MAPK/MK2 pathway, phosphorylation of sHSP25 will be induced by exposure to inflammatory mediators such as TNF and ROS. The resulting small phosphorylated sHSP25 oligomers can bind to F-actin filaments, thereby promoting increased F-actin dynamics and stress fiber formation [21], manifested by rapid reorganization of cortically localized F-actin into long transcytoplasmic fibers [35–37]. In HUVEC cells, this process was shown to be severely impaired after pharmacological inhibition of p38 MAPK [38]. Because of its unique location, the endothelial actin cytoskeleton is one of the earliest targets of ROS-mediated toxicity during an inflammatory response. In fact, it has been suggested that endothelial barrier function, which hinges on the integrity of the cytoskeleton, could be the limiting factor determining the capacity of endothelial cells to deal with oxidative stress [35]. The change in F-actin dynamics serves two main functions in the endothelium: (1) modulation of intercellular contacts [39], as well as providing a scaffold for actomyosin-based contractility to allow cell rounding and inter-endothelial gap formation [40], required for the regulated extravasation of leukocytes in response to inflammatory mediators; and (2) firmly anchor the cells to their neighbors by connecting intracellular stress fiber networks together through so-called discontinuous adherens junctions to preserve barrier function in response to stress [25,26]. Our *in vivo* results confirmed that the endothelial cell barrier in MK2-deficient liver vessels appeared to be unable to mount a proper stress fiber response, as evidenced by the absence of increased actin density and changes in the observable actin structures after TNF challenge. The failure of endothelial cells to respond appropriately to inflammatory mediators, such as TNF and TNF-induced ROS, may then result in loss of barrier integrity because of cellular damage, and excessive fluid leak, which we observed in the liver, kidneys and spleen, culminating into end-organ failure and hyperacute mortality. The speed of these events was further emphasized by the pronounced drop in body temperature, starting as early as 90–120 min after TNF challenge, indicative for microcirculatory failure of end-organs. Curiously, we only observed increased permeability for a 4 kDa tracer, while no increased albumin (70 kDa) permeability was observed. This suggests that the failure of endothelial cells to reorganize their actin cytoskeleton appeared to result mainly in increased permeability for fluid and small solutes, while uncontrolled passage of larger molecules did not occur. Contrary to our results, an earlier study reported decreased lung endothelial permeability for Evans Blue in an ovalbumin-induced asthma model [41], indicating that the response of stress-induced regulation of the cytoskeleton is highly dependent on the initial stressor and the dynamics of the model.

In order to extend our results to a more clinically relevant model of sepsis, MK2-deficient mice were subjected to CLP surgery. Also CLP-induced mortality was exacerbated in MK2-deficient mice. The reason for this increased sensitivity remains to be determined, but similar mechanisms as described for the TNF model could be involved. In addition, blocking TNF in CLP is known to actually exacerbate mortality [42]. Thus, reduced TNF levels in MK2-deficient animals because of increased instability of pro-inflammatory cytokine mRNAs could also have contributed to increased mortality.

## Conclusions

In summary, we showed that MK2-deficient mice are highly sensitized to even very low doses of TNF, leading to hyperacute mortality. ROS play an important role in this pathophysiology since the phenotype could be rescued by antioxidant treatment with tempol. In addition, the failure of endothelial cells to respond to ROS-induced toxicity with an appropriate stress fiber response, required to preserve barrier function and efficiently regulate the immune response, appeared to be involved in the phenotype. In turn, this could have led to massive edema formation, increased cellular and tissue damage, and mortality. Our results thus corroborate the dependency of actin cytoskeletal dynamics on the stress-induced p38 MAPK/MK2 pathway in an *in vivo* setting, and emphasize the importance of this pathway for stabilizing the endothelial barrier under conditions of oxidative stress. Multiple studies have highlighted the inflammation-driving role of MK2 and MK3 (reviewed in [43]) by showing that mice deficient for one or more of these kinases are protected against diverse inflammatory conditions, including arthritis, pancreatitis, skin inflammation, acute proliferative glomerulonephritis, colitis, cardiac ischemia-reperfusion injury [44], and asthma [41] or ventilator-induced [45] lung injury. Consequently, pharmacological inhibition of MK proteins has been proposed as a potential therapeutic strategy. However, our results warrant caution for the unbridled pharmacological targeting of MK2. Considering the pivotal role of TNF in many acute and chronic inflammatory conditions, systemic inhibition of MK2 could have potentially dangerous side effects at the level of endothelial barrier integrity.

## Methods

### Mice

MK2-deficient mice were generated on a C57BL/6J background, as described previously [6], and bred homozygously in our SPF facility. All mice were housed in temperature-controlled, individually ventilated cages in an SPF facility with 14/10 h light/dark cycles, food and water *ad libitum*, and used at 10–20 weeks of age. All experiments were approved by the animal ethics committee of the Faculty of Sciences of Ghent University (Belgium), performed according to its guidelines, and comply with Directive 2010/63/EU. For each experiment, mice were monitored every 30 min for the first 6 h, followed by several times daily until recovery. Moribund or surviving animals were euthanized by CO_2_ asphyxiation, followed by cervical dislocation.

### Reagents and injections

All reagents were dissolved in sterile phosphate buffered saline (PBS) and 200 μl was injected intravenously (i.v.), unless stated otherwise. Recombinant mouse TNF was produced in and purified from *Escherichia coli*, LPS content was <0.02 ng/mg (chromogenic *Limulus amebocyte* lysate assay), and was administered at various sublethal doses (10, 50, 125 or 250 μg/kg) or a lethal dose (450 μg/kg) for wild type (WT) mice. Phenol extracted *E. coli* LPS (serotype O111:B4) was purchased from Sigma (St. Louis, MO) and injected i.v. at 10 mg/kg. Tempol (Sigma) was injected intraperitoneally (i.p.) at 285 mg/kg (−45 min) and 125 mg/kg (+2.5 h). Superoxide dismutase (SOD, ICN, Aurora, OH) was injected i.p. at 3500 U/animal (−45 min) and 1800 U/animal (+2.5 h). 0.2% Evans blue (Sigma) and 50 mg/ml FITC-dextran (4 kDa, Sigma) were used to examine vascular permeability.

### Cecal ligation and puncture surgery

The CLP procedure was performed as described earlier [46]. Briefly, the mice were anesthetized using 2% isoflurane in oxygen. After shaving and disinfecting the abdomen, a 10 mm midline laparotomy was performed, followed by exposure of the cecum. Using 5–0 Ethicon suture, the cecum was ligated immediately under the ileocecal valve and subsequently perforated by a single through-and-through puncture with a 22G needle. Next, the cecum was slightly compressed until a small drop of feces appeared. The abdomen was closed in two layers, using 5–0 suture for the peritoneum and abdominal musculature, and wound clips for the skin. Following surgery, the animals were resuscitated with 1 ml 0.9% saline subcutaneously. All animals were given pre- and postoperative analgesia (Ibuprofen, 200 μg/ml in drinking water), starting 24 h before until 48 h after surgery. Mice were randomized with regard to age and genotype.

### Body temperature

Rectal body temperatures were recorded on an electronic thermometer (C28K, Comark Electronics; Littlehampton, UK).

### Vascular permeability

Animals were injected with Evans blue and FITC-dextran 30 min before dissection, followed by terminal anesthesia with ketamine/xylazine and perfusion with 50 ml heparin (Sigma) in sterile saline (5 mg/ml). Liver, kidneys, lungs, heart and spleen were dissected, minced and incubated for 24 h at 37°C in formamide (Sigma) to extract the tracers. After clearance by centrifugation, Evans blue and FITC-dextran levels were determined at 620 nm and 520 nm, respectively.

### Plasma NO_x_^−^, peroxide, and cytokine levels

EDTA plasma was prepared from blood collected via cardiac puncture after terminal anesthesia with ketamine/xylazine and immediately flash frozen in liquid nitrogen. Plasma concentrations of NO_2_^−^ and NO_3_^−^ (collectively NO_x_^−^) were determined via the Griess method as previously described [29]. Plasma peroxide equivalents were determined with a QuantiChrom Peroxide assay (DIOX-250, BioAssay Systems, CA) as per the manufacturer’s instructions. IL-1β and IL-6 levels were determined with a Bio-Plex Pro cytokine kit (Bio-Rad, Hercules, CA), as per the manufacturer’s instructions.

### Plasma hexosaminidase and lactate dehydrogenase levels

Hexosaminidase levels were determined using p-nitrophenol-N-acetyl-β-D-glucosamine substrate as described previously [47]. Lactate dehydrogenase levels were determined with a CytoTox 96 assay (Promega, Madison, WI), as per the manufacturer’s instructions.

### Immunohistochemical detection of F-actin

Livers and kidneys were dissected and fixed in 4% PFA, followed by embedding in 5% low-melting point agarose (type VIIa, Sigma). Tissue sections of 60 μm were cut on a vibratome, stained with phalloidin Alexa Fluor 488 (1/25, Invitrogen, Paisley, UK) for 20 min at RT, and counterstained with DAPI nuclear staining (1/1000, Invitrogen). Images were taken on a Zeiss LSM780 confocal microscope (Carl Zeiss Microscopy, Jena, Germany) with a 100× Plan-Apochromat/1.46 oil objective. Pixel size was 0.083 μm at an image resolution of 1024 × 1024 pixels. DAPI was excited with a Ti:Sa Laser Mai Tai (Spectra-Physics) at 790 nm and detected with a spectral bandwidth of 415–494 nm by the PMT. Alexa fluor 488 was excited using the 488 line of a Multi-Argon laser and detected with a spectral bandwidth of 499–587 nm by the Quasar detection unit. The pinhole was set at 1 Airy Unit. 80 z-sections were made with 0.34 μm z-spacing. 3D segmentation was performed using Volocity™ software, version 6.1.5 (PerkinElmer Inc., Waltham, MA). Objects were identified in a manually defined region of interest. Intensity threshold was set at 38.5% and objects smaller than 0.01 μm^3^ were discarded. For each region of interest the total number and volume of phalloidin-positive structures were determined. The number of objects per unit of volume was derived from these data, and the average size of the detected objects was calculated.

### Statistical analysis

Statistical analysis was performed with GraphPad Prism 6.03 (GraphPad Software, La Jolla, CA (USA)). Survival results were compared to each other using a log-rank (Mantel-Cox) test. For *ex vivo* analysis, WT were compared to MK2-deficient animals via one-way ANOVA with Sidak’s multiple comparisons test. Control (PBS) groups for both genetic backgrounds were pooled because they were statistically similar. Values are means ± SD.

## Abbreviations

ARE: AU-rich element
CLP: Cecal ligation and puncture
d-Gal: d-Galactosamine
FITC: Fluorescein isothiocyanate
GSH: Glutathione
HuR: Human antigen R
HUVEC: Human umbilical vein endothelial cell
IFN: Interferon
IL: Interleukin
LDH: Lactate dehydrogenase
LPS: Lipopolysaccharide
MAPK: Mitogen-activated protein kinase
MK2: MAPK-activated protein kinase 2
NO: Nitric oxide
PMT: Photomultiplier tube
ROI: Region of interest
ROS: Reactive oxygen species
sHSP: Small heat shock protein
SOD: Superoxide dismutase
SPF: Specific pathogen free
TNF: Tumor necrosis factor
TTP: Tristetraprolin
WT: Wild type
